# Pigment Formation by *Monascus pilosus* DBM 4361 in Submerged Liquid Culture

**DOI:** 10.1021/acs.jafc.5c08401

**Published:** 2025-10-08

**Authors:** Marketa Husakova, Matej Bezdicek, Barbora Branska, Karel Sedlář, Petra Patakova

**Affiliations:** † 52735 University of Chemistry and Technology, 160 00 Prague, Czechia; ‡ Division of Clinical Microbiology and Immunology, Department of Laboratory Medicine, University Hospital Brno, 625 00 Brno, Czechia; § Division of Clinical Microbiology and Immunology, Department of Laboratory Medicine, Faculty of Medicine, Masaryk University, 602 00 Brno, Czechia; ∥ Department of Biomedical Engineering, Faculty of Electrical Engineering and Communication, 48274 Brno University of Technology, 601 90 Brno, Czechia

**Keywords:** *Monascus pilosus*, pigments, catabolic repression, stress conditions

## Abstract

*Monascus pilosus* is usually cultivated on rice because of monacolin K. We focused on pigment production in submerged liquid culture (SLC) where *M. pilosus* produced different pigments compared to *M. purpureus* and *M. ruber*. From the group of classic *Monascus* pigments, there were formed mostly compounds with a five-carbon side chain, and the dominant pigment was monascuspiloin, a yellow pigment structurally similar to monascin. In SLC, previously undescribed patterns affecting pigment formation were observed, such as the Crabtree effect, carbon catabolite repression of pigments caused by glucose and other mono-/disaccharides, as well as nitrogen regulation, particularly repression of pigment formation by ammonium sulfate. The highest pigment concentration in the extract was obtained using an organic nitrogen source, specifically 340 mg/L for yellow pigments utilizing a combination of sucrose and tryptone, 346 mg/L for orange pigments using starch and tryptone, and 75 mg/L for red pigments using lactose and tryptone.

## Graphical abstract

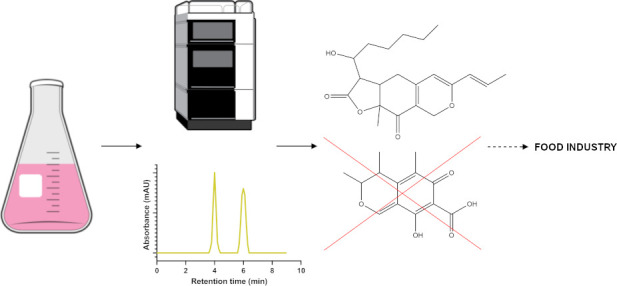

## 1 Introduction

The *Rubri* section of the genus *Monascus* can be phylogenetically divided into two clades: *M. purpureus* clade and *M. pilosus*-*M. ruber* clade, which differ in the presence of specific biosynthetic gene clusters.^1^ Until now, most research articles dealing with *Monascus* pigment production focused on different strains of *Monascus purpureus*. However, this species produces high levels of pigments, frequently together with the mycotoxin citrinin.^2^ On the contrary, a representative of the second clade, *M. pilosus*, is mainly studied for its biosynthesis of monacolin K,^1^ a statin that inhibits the key enzymatic step in cholesterol biosynthesis, which, however, is produced under different conditions than pigments.^1,3^

In the European Union, the use of *Monascus* fungus was originally permitted only in the form of food supplements (red yeast rice (RYR) fermented by *Monascus purpureus*, which provides 10 mg per day intake of monacolin K) with the health claim “Monacolin K from red yeast rice contributes to the maintenance of normal blood cholesterol levels”.^4,5^ However, this health claim was probably worded incorrectly, given the differences in secondary metabolite profiles between *M. purpureus* and *M. pilosus*/*M. ruber*, and suggests earlier ambiguous identification of species/strains of the genus *Monascus*. In addition, during use of the food supplement with RYR, and adverse effects of monacolin K on the musculoskeletal system and liver, which were reported in some cases with intake of monacolin K as little as 3 mg from RYR/day,^6^ the European Commission amended Regulation (EU) No 432/2012 (issued as the Commission Regulation (EU) 2022/860).^7^ It decided that products based on RYR shall provide less than 3 mg of monacolins in a daily dose and must be appropriately labeled.^7^ The American Food and Drug Administration (FDA) never permitted food supplements with monacolin K but approved Ankascin 568 ^8^ as a new dietary ingredient free of both citrinin and monacolin K. Ankascin 568 contains yellow pigments, monascin (Figure 1A) and ankaflavin, and should lower cholesterol levels.^9^

**Figure 1:**
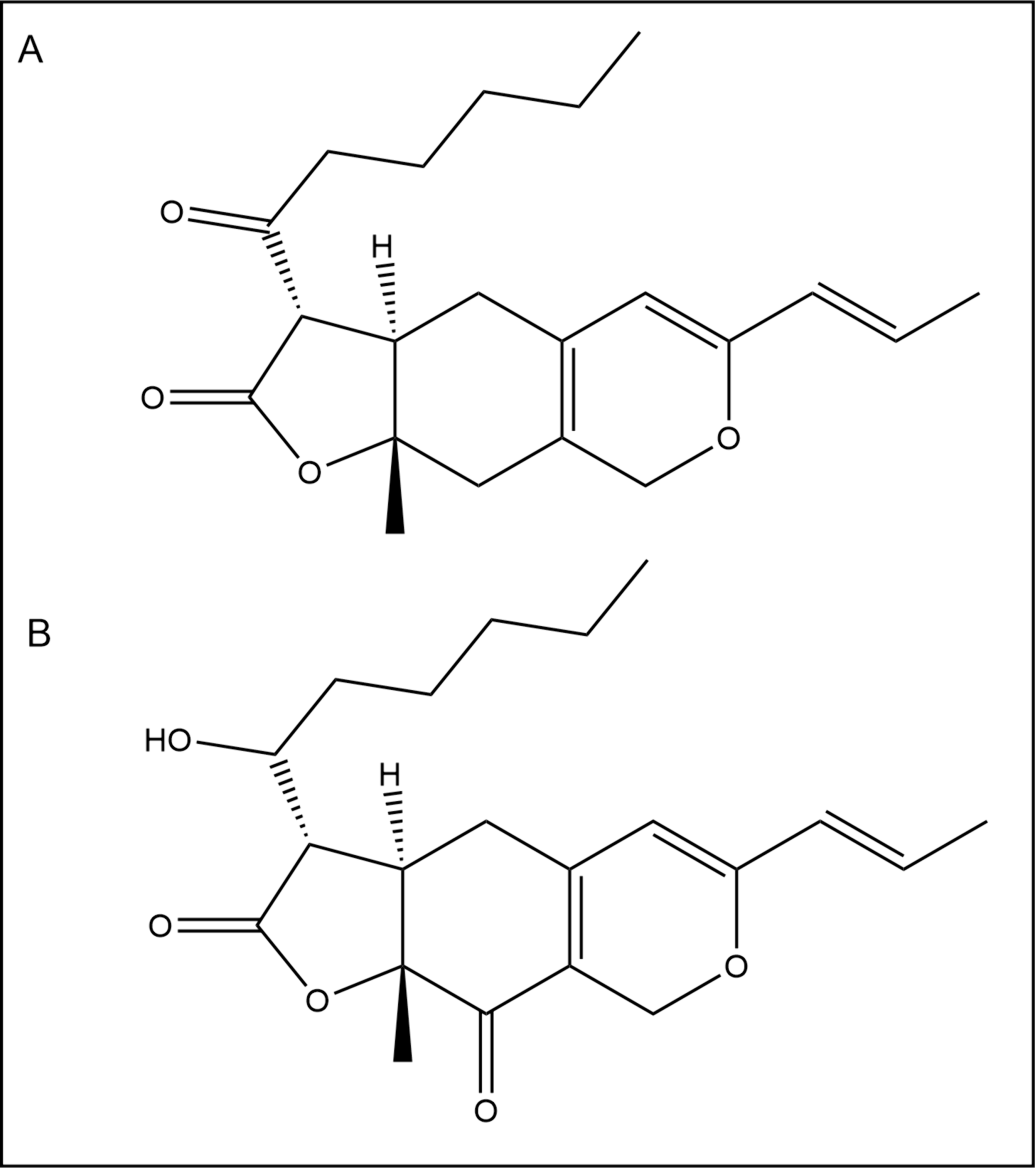
Chemical structures of main yellow *Monascus pilosus* pigments (monascin (A) monascuspiloin (B)).

The levels of pigments produced by *M. pilosus* are lower compared to *M. purpureus* strains and the production profile significantly differs, with monascuspiloin (Figure 1B) being the main yellow pigment.^10,11^ Monascuspiloin (known also as monascinol) is probably formed from monascin by reduction of the C-3′ ketone to the corresponding alcohol.^12^ Monascuspiloin was suggested as a cytotoxic agent against human prostate cancer cells,^13,14^ as well as a potential anti-inflammatory^15^ and (photo)­antimicrobial agent.^11^

An advantage of *M. pilosus* is its production of pigments without contamination by mycotoxin citrinin, thus having the potential for safe use in the food industry. However, an increase in pigment production should be achieved. In this study, the strain *Monascus* sp. DBM 4361 was first reidentified as *M. pilosus* and then was cultivated under osmotic stress culture conditions with the goal to increase pigments production in submerged liquid culture (SLC). Osmotic stress induced by higher glucose/sucrose and/or NaCl concentrations was selected as a factor that could lead to this goal based on previous findings for *M. purpureus* ^2,16–18^ and *M. ruber*,^19–21^ because there is no relevant information for *M. pilosus*. Nevertheless, to our surprise, glucose was not found to be a good carbon source, suggesting potential carbon catabolite repression (CCR) of pigments formation by glucose. The CCR regulatory mechanism is usually associated with the rational sequential utilization of mono- and disaccharides in bacteria and fungi. However, it has been demonstrated, for example in the model organism *Aspergillus nidulans*,^22^ that CCR influences various physiological processes, mycelial development, and secondary metabolism via carbon metabolism. The CCR control of secondary metabolites formation was observed for, aflatoxin production by *Aspergillus flavus*,^23^ penicillin production by *Penicillium chrysogenum*,^24^ citrinin production by *Penicillium citrinum* ^25^ and lovastatin production by *Aspergillus terreus* ^26^ or *M. pilosus*.^27^ To confirm or disprove the glucose CCR hypothesis and to identify conditions resulting in high yields of pigments, combinations of different carbon and nitrogen sources were screened. This led to the first mapping of the conditions affecting *M. pilosus* pigment production during SLC.

## 2 Materials and Methods

### 2.1 Microbial Strain

The strain *Monascus* sp. DBM 4361, originally isolated from functional red yeast rice, was used in this study. The strain was deposited in the Culture Collection of the Department of Biochemistry and Microbiology (DBM), University of Chemistry and Technology Prague. *Monascus purpureus* NBRC 4482 and *Monascus pilosus* NBRC 4480, purchased from the Biological Resource Center (NBRC, Japan) were used as reference only. All strains were maintained on Sabouraud agar slants (VWR Chemicals) at 4 °C. Inoculum suspension (conidia) was prepared from grown mycelia (5 days) on agar slants. The surface was washed with physiological solution (0.9% NaCl) containing 0.01% of Tween 80 and scraped with a sterile inoculation loop.

### 2.2 ITS Region Sequencing and Identification of the Strain

Strain identification was achieved through sequence analysis of the ITS1, 5.8S, and ITS2 regions of rDNA. This region was amplified using a universal panfungal primer pair designed by Ferrer et al.^28^ and subjected to Sanger sequencing in *Monascus* sp. DBM 4361 and additionally in *Monascus purpureus* DBM 4360. For direct comparison, type strains *Monascus purpureus* NBRC 4482 and *Monascus pilosus* NBRC 4480, were included in the analysis. Additionally, other *Monascus* sp. sequences were obtained from the NCBI RefSeq database, particularly ITS from the Fungi type and reference material database.^29^ Sequence alignment was performed using MEGA X software^30^ using the ClustalW algorithm.^31^ The final phylogenetic tree was constructed in MEGA X by the maximum likelihood method and the Tamura-Nei substitution model.^32^

### 2.3 Submerged Cultivation Conditions

#### 2.3.1 Cultivation under Stress Conditions

Cultivation under osmotic stress was performed in a 250 mL Erlenmeyer flask containing 100 mL of culture medium. The culture medium composition was (g/L): KCl 0.5; KH_2_PO_4_ 4; ZnSO_4_·7H_2_O 0.01; MgSO_4_·7H_2_O 0.5; FeSO_4_·7H_2_O 0.01 and combinations of carbon and nitrogen sources stated in Table 1. Osmotic stress was induced by higher carbon sources (glucose/sucrose) concentrations (50, 100, 150 g/L) and/or by addition of sodium chloride (5–150 g/L) or glycerol (0.5–1M) (see Table 1). The initial pH was adjusted to 5.5. Culture medium was inoculated with the spore suspension (1% v/v) and all cultivations were carried out on a rotary shaker (100 rpm) at 30 °C for 14 days.

**Table 1:** Concentrations of Glucose/Sucrose, Nitrogen Source, and Sodium Chloride in Stress Cultivation Media

code	carbon source (g/L)	nitrogen source (g/L)	sodium chloride (g/L)
A (control)	glucose 50	ammonium sulfate 5	0
A50+5	glucose 50	ammonium sulfate 5	5
A50+20	glucose 50	ammonium sulfate 5	20
A50–35	glucose 50	ammonium sulfate 5	35
A50+50	glucose 50	ammonium sulfate 5	50
A50+70	glucose 50	ammonium sulfate 5	70
A50+100	glucose 50	ammonium sulfate 5	100
A50+125	glucose 50	ammonium sulfate 5	125
A50+150	glucose 50	ammonium sulfate 5	150
A100	glucose 100	ammonium sulfate 5	0
A100+35	glucose 100	ammonium sulfate 5	35
A100+50	glucose 100	ammonium sulfate 5	50
A100+70	glucose 100	ammonium sulfate 5	70
A100+100	glucose 100	ammonium sulfate 5	100
A150	glucose 150	ammonium sulfate 5	0
B (control)	sucrose 50	tryptone 8.54	0
B50+50	sucrose 50	tryptone 8.54	50
B100	sucrose 100	tryptone 8.54	0

After cultivation, the grown mycelia were separated from culture broth, weighed and a part of the mycelium was used for extraction and determination of *Monascus* pigments. The pH of culture broth was determined.

#### 2.3.2 Cultivation Using Different Carbon and Nitrogen Sources

Cultivations with different carbon and nitrogen sources were performed as described in Husakova et al.^2^ Briefly, cultivation medium contained KCl (0.5 g/L), KH_2_PO_4_ (4 g/L), (ZnSO_4_·7H_2_O (0.01 g/L), MgSO_4_·7H_2_O (0.5 g/L), FeSO_4_·7H_2_O (0.01g/L), carbon sources (g/L): glucose 50; rice starch 50; fructose 50; maltose 47.5; sucrose 47.5; glycerol 51.2; lactose 47.5; arabinose 50; xylose 50; and nitrogen sources (g/L): casamino acids 8.55; monosodium glutamate 14.10; ammonium sulfate 5; peptone 8.55; sodium nitrate 6.41; yeast extract 8.54; tryptone 8.54; ammonium chloride 4.27. The concentrations used were calculated to reach the same carbon and nitrogen quantity. Cultivations were performed in 24-well microplates at a final volume of 2 mL, and all sources were used in combinations of each carbon source with each nitrogen source. Initial pH was adjusted to 5.5. Culture medium was then inoculated with the inoculum suspension (1% v/v) and all cultivations were carried out in triplicate on a rotary shaker (100 rpm) at 30 °C for 14 days.

After cultivation, the grown mycelia were separated from the culture broth and were used for extraction and determination of pigments.

### 2.4 Extraction and Determination of *Monascus* Pigments

Grown mycelia were extracted in the ratio 0.1 g of wet biomass:1 mL of extraction solution on a rotary shaker at 30 °C for 40 min. As an extraction solution, 85% acidified (pH 4) ethanol was used.

Extracts were then analyzed by UltraHigh-Performance Liquid Chromatography (Agilent Technologies 1260 Infinity II). UHPLC analysis was performed utilizing the protocol published in Husakova et al*.* ^11^ UV–vis absorption spectra of individual compounds separated by UHPLC were used for their identification because the spectra (in range 200–600 nm) are specific for yellow, orange and red *Monascus* pigments.^11^ Yellow pigment monascin (Sigma-Aldrich) and orange pigment rubropunctatin (1717 CheMall Corporation) were purchased as commercially available substances. Rubropunctamine was prepared from the rubropunctatin standard by reaction with NH_4_OH (Penta) in 80% ethanol. These were used for the calibration. Unknown yellow, orange and red pigments, as well as monascuspiloin, were identified on the basis of their absorption spectra and quantified as equivalents to their respective standards, i.e., monascin, rubropunctatin and rubropunctamine.

### 2.5 Determination of Carbon Source Utilization and Production of Primary Metabolites

An HPLC (Agilent Technologies 1200 Infinity) device with a refractometric detector was used to quantify utilization of glucose and the production of primary metabolites. As primary metabolites, ethanol, glycerol, and organic acids (malic, succinic and fumaric acid) were determined. Pure compounds were used as standards. The analyses were performed under the following conditions: Watrex 250 × 8 mm Polymer IEX H+ 8 μm column; mobile phase, 5 mM H_2_SO_4_ in demineralized water; isocratic elution at a flow rate 1 mL/min at 60 °C; injection volume 20 μL; analysis run for 16 min.

### 2.6 Statistical Analysis

All cultivation tests were performed in triplicate. One-way ANOVA analysis with Dunnett’s multiple comparisons test was performed for osmotic stress cultivation data. The control cultivations data with glucose or sucrose as carbon sources (“A” and “B”) were compared to those obtained under the corresponding stress conditions (see Table 1). One-way ANOVA analysis with Tukey’s multiple comparisons test was performed for cultivation using different carbon and nitrogen sources data. The data obtained under these conditions were compared with each other. Statistical analysis was performed using GraphPad Prism software. Significance levels were set up as *p < 0.05; **p < 0.01; ***p < 0.001.

## 3 Results

### 3.1 Identification and Phylogeny

Newly sequenced ITS1, 5.8S, and ITS2 regions for *Monascus* sp. DBM 4361 and *Monascus purpureus* DBM 4360 have been deposited in the DDBJ/ENA/GenBank under the accessions PV803112 and PV803113, respectively. First, *Monascus* sp. DBM 4361 was identified to the species level. A region of rDNA, including the ITS1, 5.8S, and ITS2 regions, was amplified for each strain and compared with reference sequences published in the NBRC Online Catalogue. For direct comparison, strains obtained from the NBRC collection, *M. pilosus* NBRC 4480 and *M. purpureus* NBRC 4482 were included in the analysis. *Monascus* sp. DBM 4361 was identified as *Monascus pilosus*, having a 100% identical sequence as *Monascus pilosus* NBRC 4480 Sequence ID IF00448001. Additional *Monascus* sp. ITS1, 5.8S, and ITS2 from the RefSeq database were added into the analysis to infer phylogenetic relationships within the genus, see also Figure 2.

**Figure 2:**
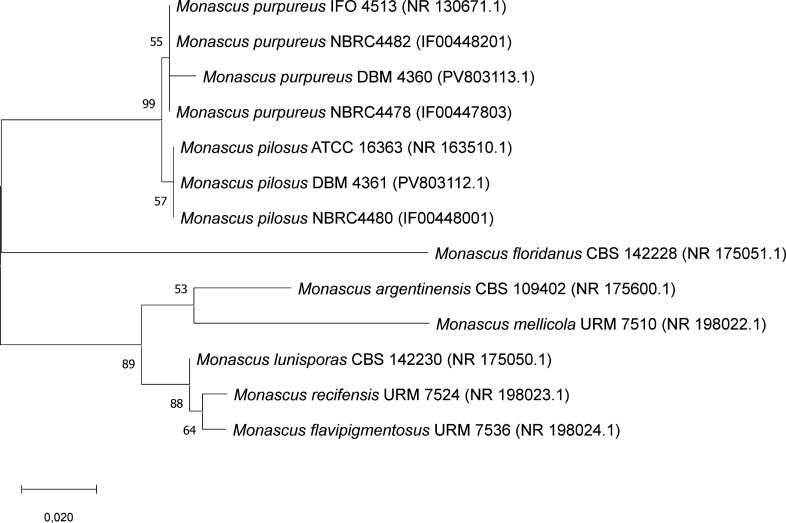
Evolutionary relationships among *Monascus* sp. inferred by the Maximum Likelihood method and the Tamura-Nei model. The tree with the highest log likelihood (−2162,30) is shown. The percentage of trees in which the associated taxa clustered together, calculated form 500 bootstrap replicates, is shown next to the branches. The tree is drawn to scale, with branch length sequences. In the final data set were a total of 798 positions. Evolutionary analyses were conducted in MEGA X. Accession numbers of sequences used in the analysis are written in brackets with IF* corresponding to the NBRC online catalogue and the rest to the NCBI RefSeq/GenBank databases.

### 3.2 Effects of Osmotic Pressure on Fungal Growth and Production of Primary and Secondary Metabolites

The strain *Monascus* sp. DBM 4361, newly specified as *M. pilosus*, was subjected to a screening experiment mapping the effect of osmotic stress on primary and secondary metabolism. As a control, cultivation with glucose (50 g/L), in combination with ammonium sulfate (5 g/L), was used. In selected cases, sucrose was also used in combination with tryptone. The osmotic pressure, induced by the addition of salt, and/or a higher concentration of carbon source, inhibited growth. This was observed as a decrease in wet biomass weight and incomplete utilization of the carbon source (see Figure 3). Total growth suppression was reached only in the case of salt at 125 and 150 g/L NaCl. Higher concentrations of saccharides, up to 150 g/L glucose without NaCl addition, did not inhibit growth.

**Figure 3:**
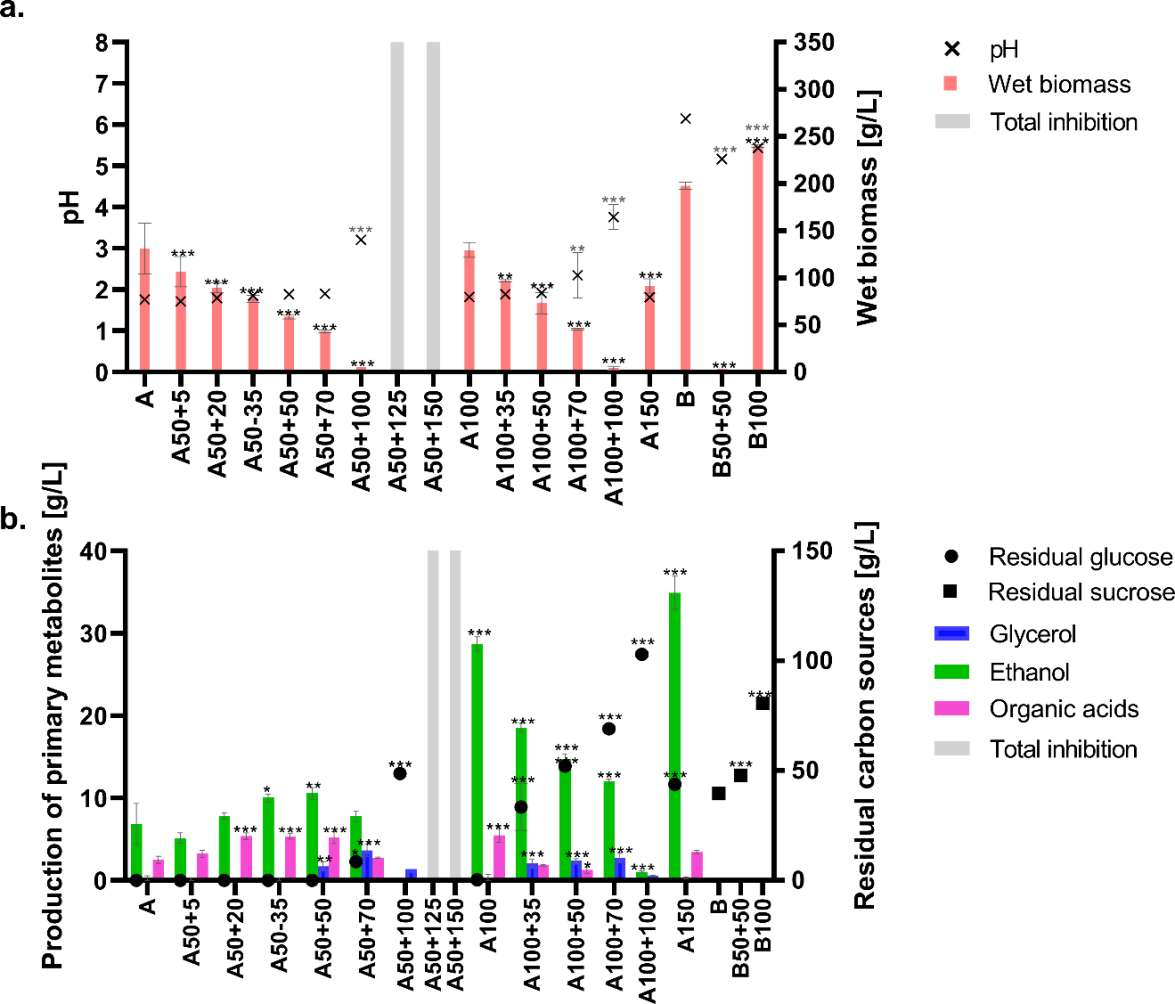
Wet biomass, pH (a), and production of primary metabolites and consumption of carbon sources (b) were determined under stress conditions (for explanation of codes see Table 1); A and B were control cultures for glucose and sucrose, respectively. All cultivations were performed in triplicate, and data are presented as a mean of three values with bars representing standard deviations statistical significance: *p < 0.05; **p < 0.01; ***p < 0.001.

The effects of osmotic pressure on primary metabolite production are shown in Figure 3b. Production of ethanol under aerobic conditions in the presence of glucose was observed in almost all cases and the highest ethanol concentrations (28–35 g/L) were determined in the excess of glucose (100–150 g/L). Higher osmotic stress (concentrations of glucose of 50 g/L + NaCl 50 g/L or higher) led to the production of glycerol at concentrations around 3 g/L, but under other conditions, glycerol production was not detected. Production of organic acids (citric, malic, succinic and fumaric acids), as in the case of ethanol, was detected only in the presence of glucose, and resulted in a decrease in pH of the culture broth.

With regard to pigment production, it has been shown that osmotic stress—mild in the case of glucose (i.e., 5 g/L NaCl) or more severe in the case of sucrose (i.e., 50 g/L NaCl or 100 g/L sucrose without salt)—was beneficial for pigment production, see Figure 4. Nevertheless, only yellow pigments were detected in the mycelium extract. Specifically, monascuspiloin and monascin were present for both carbon sources, while unidentified yellow pigment(s) were detected only in sucrose-containing culture medium (see chromatograms in the Supplementary Figure S1). Under a wide range of selected culture conditions, including those that resulted in relatively good biomass production (glucose concentration 100 g/L without salt or glucose concentration of 100 g/L together with 35 g/L NaCl), pigments were not formed at all or only in negligible amounts. With increasing NaCl concentrations, monascuspiloin concentrations decreased and monascin became the major/only pigment detected.

**Figure 4:**
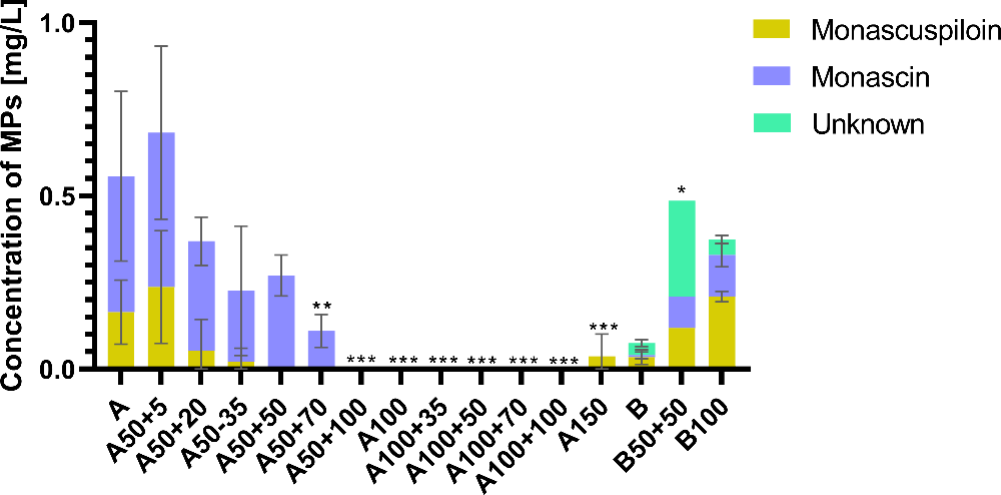
Concentrations of pigments determined under stress conditions (for explanation of codes, see Table 1); A and B were control cultures for glucose and sucrose, respectively. All cultivations were performed in triplicate, and data are presented as a mean of three values with bars representing standard deviations, statistical significance: *p < 0.05; **p < 0.01; ***p < 0.001.

### 3.3 Effects of Different Combinations of Carbon and Nitrogen Sources on the Production of *Monascus* Pigments

Since the results obtained under stressful conditions revealed unexpected behavior and indicated catabolic repression of pigment formation during glucose utilization, we decided to perform a broader mapping of pigment formation using various combinations of carbon and nitrogen sources. Final concentrations of pigments extracted from biomass are summarized in Figure 5 (yellow pigments), Table 2 (orange pigments) and Figure 6 (red pigments), and data for biomass produced under particular conditions are shown in Figure S2. The highest amounts of pigments were obtained using arabinose, starch, sucrose, glycerol, and lactose as carbon sources; in the case of nitrogen sources, complex sources, such as tryptone, and yeast extract, proved to be the most effective, followed by casamino acids, peptone, and in some cases, the inorganic source, sodium nitrate. It was confirmed that neither glucose nor ammonium sulfate are good carbon and nitrogen sources respectively for pigment production. The use of arabinose appears to be very interesting, as it led to the production of pigments, unlike xylose, the other pentose carbohydrate tested. Furthermore, as in the previous test, the strain was found to produce mainly yellow pigments.

**Figure 5:**
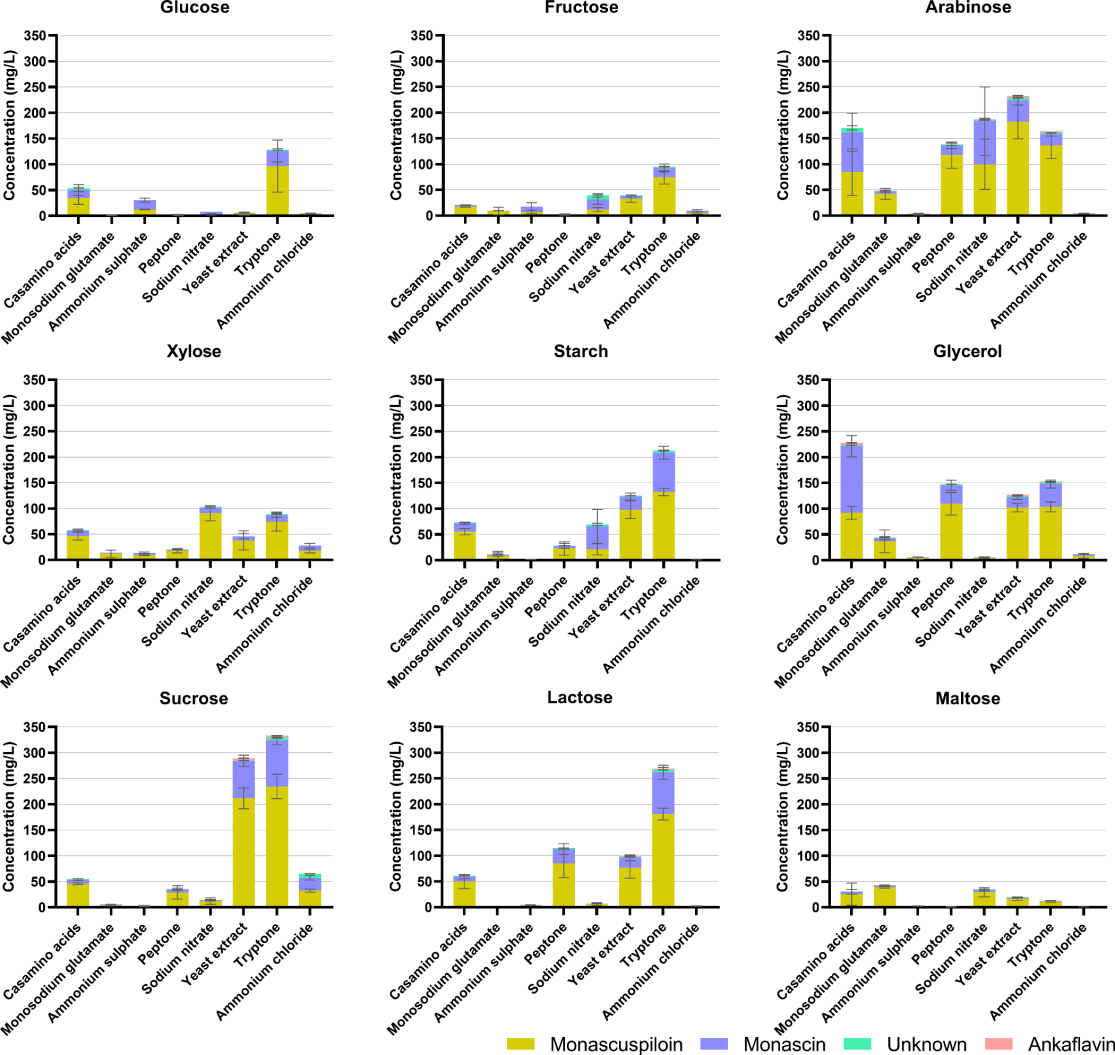
Production of yellow pigments under different cultivation conditions. All cultivations were performed in 24 well microcultivation plates, in three parallels. The data are presented as a mean of three parallels, with bars representing standard deviations. Statistical significance is stated in Supplementary Table S2.

**Table 2:** Production of Orange Pigments under Different Cultivation Conditions^a^

	mg/L			
carbon and nitrogen source	rubropunctatin	monascorubrin	unknown pigment	sum of orange pigments
glucose + casamino acids	10.2			10.2
glucose + (NH_4_)_2_SO_4_	1.5			1.5
glucose + tryptone	6.1			6.1
starch + NaNO_3_	52.8		5.1	57.9
starch + tryptone	173.5	6.1		179.6
fructose + (NH_4_)_2_SO_4_	0.9		2.1	3.0
fructose + NaNO_3_	38.3		19.1	57.4
fructose + tryptone	3.8			3.8
maltose + casamino acids	1.6		2.7	4.3
sucrose + NaNO_3_	3.9	0.8		4.7
sucrose + tryptone	50.7	2.3	3.1	56.1
glycerol + casamino acids	8.5			8.5
glycerol + tryptone	25.5	1.8		27.3
lactose + tryptone	20.1		2.1	22.2
arabinose + casamino acids	277.3		68.9	346.2
arabinose + NaNO_3_	3.5		1.6	5.1

a All cultivations were performed in 24 well microcultivation plates in three parallels. The data are presented as a mean of three parallels. Statistical significance is stated in Supplementary Table S2.

**Figure 6:**
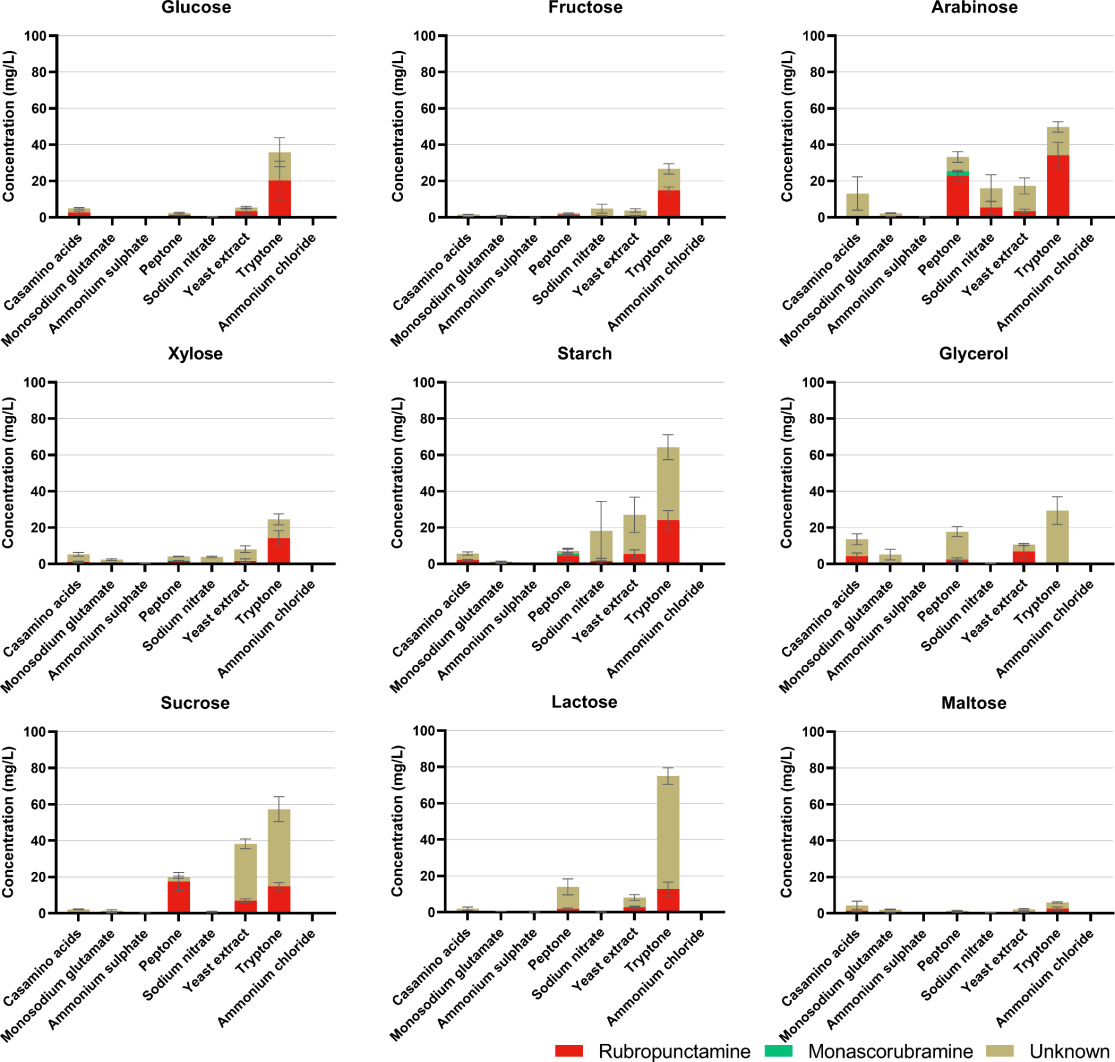
Production of red pigments under different cultivation conditions. All cultivations were performed in 24-well microcultivation plates in three parallels. The data are presented as a mean of three parallels, with bars representing standard deviations. Statistical significance is stated in Supplementary Table S2.

The most abundant yellow pigments were monascuspiloin and monascin (see Figure 5), which were detected under all cultivation conditions. An unknown yellow pigment was detected under certain culture conditions, having a lower concentration compared to monascuspiloin and monascin. Ankaflavin, the seven carbon side chain analogue of monascin, was detected only under certain cultivation conditions and at lower concentrations compared to monascuspiloin/monascin (1:0.03; 1:0.10); the production ratio of monascuspiloin to monascin varied from 1:06 (combination of maltose with tryptone) to 1:3.5 (combination of fructose with peptone), see Supplementary Table S1. The impact of carbon/nitrogen source on the abundance of individual pigments was observed; in some cases (glycerol with casamino acids, starch or fructose with sodium nitrate), monascin became the most abundant yellow pigment.

The production of orange pigments was detected only under selected culture conditions (see Table 2), and the range of specified concentrations was wide. The best sources for orange pigment production were arabinose in combination with casamino acids, starch in combination with tryptone, or sodium nitrate in combination with fructose. With xylose in combination with any nitrogen source, no orange pigments were detected; the use of glucose, glycerol, lactose and maltose, in only some cases, resulted in small pigment yields. The most abundant orange pigment was rubropunctatin; its seven-carbon side chain analogue – monascorubrin was only detected in the presence of tryptone (the average ratio of rubropunctatin to monascorubrin was 1:0.04, see Supplementary Table S1). Moreover, unknown orange pigments were detected under certain conditions, and at low concentrations.

The chemical conversion of orange pigments to red occurred under suitable conditions, nonacidic pH and the availability of compounds with a free amino group. Therefore, the highest concentrations of red pigments were detected with organic nitrogen sources (tryptone, yeast extract, peptone) and surprisingly, in combination with some carbon sources (starch or arabinose) and nitrate (see Figure 6) where the pH was above 4 (Supplementary Figure S3). Next to rubropunctamine and monascorubramine, some unknown red derivatives were detected. The average ratio of rubropunctamine to monascorubramine was 1:0.14, see Supplementary Table S1.

## 4 Discussion

Based on previously determined pigment production profiles,^10,11^ it was expected that *Monascus* sp. DBM 4361 was a species of *M. pilosus*, which was clearly confirmed by comparing sequences for the ITS1, 5.8S and ITS2 regions of rDNA with sequences of type strains *M. purpureus* NBRC 4482 and *M. pilosus* NBRC 4480. The main pigment produced by the strain *M. pilosus* DBM 4361 was monascuspiloin, a yellow analogue of monascin with a hydroxyl group instead of a carbonyl group. The major unidentified yellow pigment of *M. pilosus* DBM 4361 with a retention time of 5.9 min (see Figure S1) might be monasfluor A^33,34^ based on its fluorescence emission and typical absorbance spectrum corresponding to yellow pigment^11^ (data not shown). Other unknown yellow pigments might be those previously detected in the *M. pilosus* species, i.e., monapilosusazaphilone or monascusazaphilol,^35–37^ however future confirmation is necessary. As for the unidentified red pigments (see Figure 6), these are probably the result of a chemical reaction between orange rubropunctatin and amino acids or peptides sourced from complex nitrogen sources, tryptone, yeast extract, or peptone.^38^

Under the same culture conditions, *M. purpureus* DBM4360 produced both five- and seven-carbon side chain analogues (i.e., monascin and ankaflavin, rubropunctatin and monascorubrin, and rubropunctamine and monascorubramine).^2^ In silico analysis of available genomic data^1^ showed that the biosynthetic pathways for pigment production in *M. purpureus* and *M. pilosus* differ by the insertion of seven nonessential genes into the biosynthetic cluster for pigment production in *M. pilosus*. However, the genes for the production of both 5- and 7-carbon fatty acids, *fasA* and *fasB* (also known as *MrpigJ* and *MrpigK*) were found in all *M. pilosus* genomes including *M. pilosus* BCRC 38072 ^39^ as in *M. purpureus*. Unfortunately, four published genomes of different strains of *M. pilosus* MS-1, YDJ-1, YDJ-2, and K104061 available in GenBank are not annotated, and only the biosynthetic pathways of monacolin K, not pigments, have been analyzed in detail so far.^40^ Nevertheless, it has recently been experimentally confirmed that knockout of the *pigA* gene, responsible for the biosynthesis of the main pigment skeleton in the *Monascus* genus, leads to a dramatic decrease in pigment production in *M. pilosus* MS-1.^41^

Although stress conditions were expected to stimulate pigment production,^17–19,42^ increasing concentrations of NaCl and glucose actually led to a decreasing trend in pigment synthesis. The production of only yellow pigments in very low concentrations led us to hypothesize that glucose/sucrose induced catabolite repression of secondary metabolites (pigments). Confirmatory small-scale cultivations indicated potential carbon catabolite repression (CCR) in the presence of most monosaccharides (except arabinose) and the disaccharide maltose. It seems that the CreA transcriptional repressor, the key CCR active element, actively inhibits the transcription of secondary metabolite genes in the presence of glucose or other mono- and disaccharides in cooperation with other *cre* gene products, and plays a fundamental role in this regulation.^23^ Although CCR is a well-known phenomenon,^43^ it is usually associated with the formation of enzymes and details of how this type of regulation works, particularly in fungi, have not yet been elucidated in detail.^44^ In simplified terms it is hypothesized that glucose, after its uptake into the cell and subsequent phosphorylation, serves as a signal for CreA phosphorylation and translocation to the nucleus. In the nucleus, phosphorylated CreA can then bind to a specific binding site SYGGRG and directly inhibit the transcription of certain gene clusters, including clusters for the biosynthesis of secondary metabolites.^45^ The glucose repression of lovastatin (monacolin K) production in *M. pilosus* ^27^ seems similar to our results and was also attributed to CCR. In addition, in the case of *M. purpureus* YY-1,^46^ *creA* gene was repressed in rice (starch-containing) medium, which also correlates with our results; good pigmentation occurred using starch as a carbon source in this and in previous studies.^10,11^ However, CCR involvement in the *M. pilosus* pigments formation still requires future confirmation.

Nevertheless, another factor that contributes to metabolic flux and to the availability of precursors for the biosynthesis of secondary metabolites (especially acetyl-CoA) is the rate of transport of individual carbohydrates into cells. This phenomenon, not governed by CreA, has been described in *Aspergillus niger* grown in a mixture of saccharides,^47^ and could also be partly responsible for differences in pigment production when using different carbohydrates.

Glycerol as an osmoprotectant was formed only with the addition of NaCl. Furthermore, production of ethanol in the presence of glucose was detected. *Monascus* fungi have a respiro-fermentative metabolism and often exhibit the Crabtree effect in a glucose-rich medium,^48^ favoring fermentation and ethanol production, even under aerobic conditions. Respiratory metabolism generates more energy in the form of ATP and produces precursors, mainly acetyl-CoA and malonyl-CoA for pigments, citrinin and fatty acid biosynthetic pathways. Thus, it seems that one of the key factors for good pigment production is sufficient oxygen transport to all filaments in the mycelium. This is influenced in submerged liquid culture (SLC) by fungal morphology, where the preferred growth is either filamentous or in small size pellets.^49^ The predominance of fermentative metabolism, under the conditions of saccharide excess, in *M. pilosus*, manifested by ethanol production, may also have been one of the reasons for suppression of pigment formation. *Monascus* fungi can also utilize the ethanol produced as a carbon source,^50^ but the condition for this use is the lack of a carbohydrate carbon source. Thus, the use of ethanol as a carbon source was probably only a marginal phenomenon in our experiments. However, the addition of ethanol into submerged cultivation led to an effect on the pigment biosynthetic pathway and an increasing production of yellow pigments.^51^ Therefore, a potential link between ethanol production and production of mainly yellow pigments by our strain *M. pilosus* is possible.

Surprisingly, screening tests of various combinations of carbon and nitrogen sources also revealed relatively strong repression of pigment formation when ammonium sulfate was used. Nitrogen regulation of fungal secondary metabolites is known to be governed by the AreA transcriptional regulator^52^ and it was shown that this regulator was involved in *M. ruber* pigment production.^53^ Usually, an excess of nitrogen limits the production of secondary metabolites, as do easily usable nitrogen sources such as ammonium salts.^52^ Nevertheless, the formation of various secondary metabolites is promoted or inhibited by different forms of nitrogen sources; aflatoxin production by *A. flavus* ^54^ was promoted by ammonium ions and repressed by nitrate but the opposite was true for sterigmatocystine production by *A. nidulans*.^55^ High ammonium chloride (15 g/L) resulted in repression of pigment biosynthesis^56^ but 5 g/L of ammonium chloride (similar concentration as in our case) resulted in yellow pigment overproduction in a different strain of *M. purpureus*.^57^

If we compare the strains *Monascus pilosus* DBM 4361 with *Monascus purpureus* DBM 4360,^2^ *M. pilosus* produced 5–10× less pigments (under similar culture conditions using the same methodology for pigment determination), and it predominantly produced yellow pigments in contrast to *M. purpureus*, which produced mainly orange and red pigments. Furthermore, there was a striking difference in that *M. pilosus* DBM 4361 produced almost exclusively pigments with a 5-carbon side chain, unlike *M. purpureus* DBM 4360,^2^ where a constant ratio between 5- and 7-carbon pigment analogues was maintained under various cultivation conditions. There were also differences in the preference for carbon and nitrogen sources and in their combinations that led to the production of pigments. Glucose, fructose, xylose or maltose, which were poor carbon sources for *M. pilosus* pigment production, were not so for *M. purpureus*, as well as nitrogen sources – ammonium sulfate or ammonium chloride. This may be based on the fact that nitrogen in different forms may lead to differing regulation of secondary metabolite production in different fungal species.^52^

This article provides information on the production of pigments using *M. pilosus* under submerged conditions, with monascuspiloin identified as the main pigment under almost all cultivation conditions. It also points out not only the differing production profiles of pigments, but also the different regulations of secondary metabolite production in *M. pilosus* and *M. purpureus*. Unlike *M. purpureus*, *M. pilosus* does not produce the mycotoxin citrinin, which gives it a significant advantage in both food production and the production of biologically active substances.

## Supplementary Material







## Data Availability

The sequences of ITS1, 5.8S, and ITS2 regions for *Monascus pilosus* DBM 4361 and *Monascus purpureus* DBM 4360 have been deposited in the DDBJ/ENA/GenBank under accessions PV803112 and PV803113, respectively.

## Notes

Additional information (typical chromatograms of secondary metabolites including identification of individual peaks) and other evaluated data used in the paper, namely, concentration of wet biomass after cultivation and final pH during cultivation on individual carbon sources (PDF)
Ratios of individual pigments (XLSX)
Statistical significance (ordinary one-way ANOVA with Dunnett's multiple comparisons test and ordinary one-way ANOVA with Tukey's multiple comparisons test) (XLSX)
