# Supplementary material for: Pigment Formation by Monascus pilosus DBM 4361 in Submerged Liquid Culture
Source: J Agric Food Chem. 2025 Oct 8;73(42):26900–9. doi: 10.1021/acs.jafc.5c08401 (PMC12550855; doi:10.1021/acs.jafc.5c08401)
Supplement: Supplementary file 1 [file jf5c08401_si_001.pdf]

Pigment formation by *Monascus pilosus* DBM 4361 in submerged liquid culture

Marketa Husakova[1] https://orcid.org/0000-0001-6120-307X, Matej Bezdicek[2, 3] https://orcid.org/0000-0002-5833-8325, Barbora Branska[1] https://orcid.org/0000-0001-6536-7063, Karel Sedlář[4] https://orcid.org/0000-0002-8269-4020, Petra Patakova[1,*] https://orcid.org/0000-0002-9410-4454

[1] *University of Chemistry and Technology, Prague 160 00, Czechia*

[2] *Division of Clinical Microbiology and Immunology, Department of Laboratory Medicine, University Hospital Brno, Brno 625 00, Czechia*

[3] *Division of Clinical Microbiology and Immunology, Department of Laboratory Medicine, Faculty of Medicine, Masaryk University, Brno 602 00, Czechia*

[4] *Department of Biomedical Engineering, Faculty of Electrical Engineering and Communication, Brno University of Technology, Brno 601 90, Czechia*

[*] Corresponding author: petra.patakova@vscht.cz

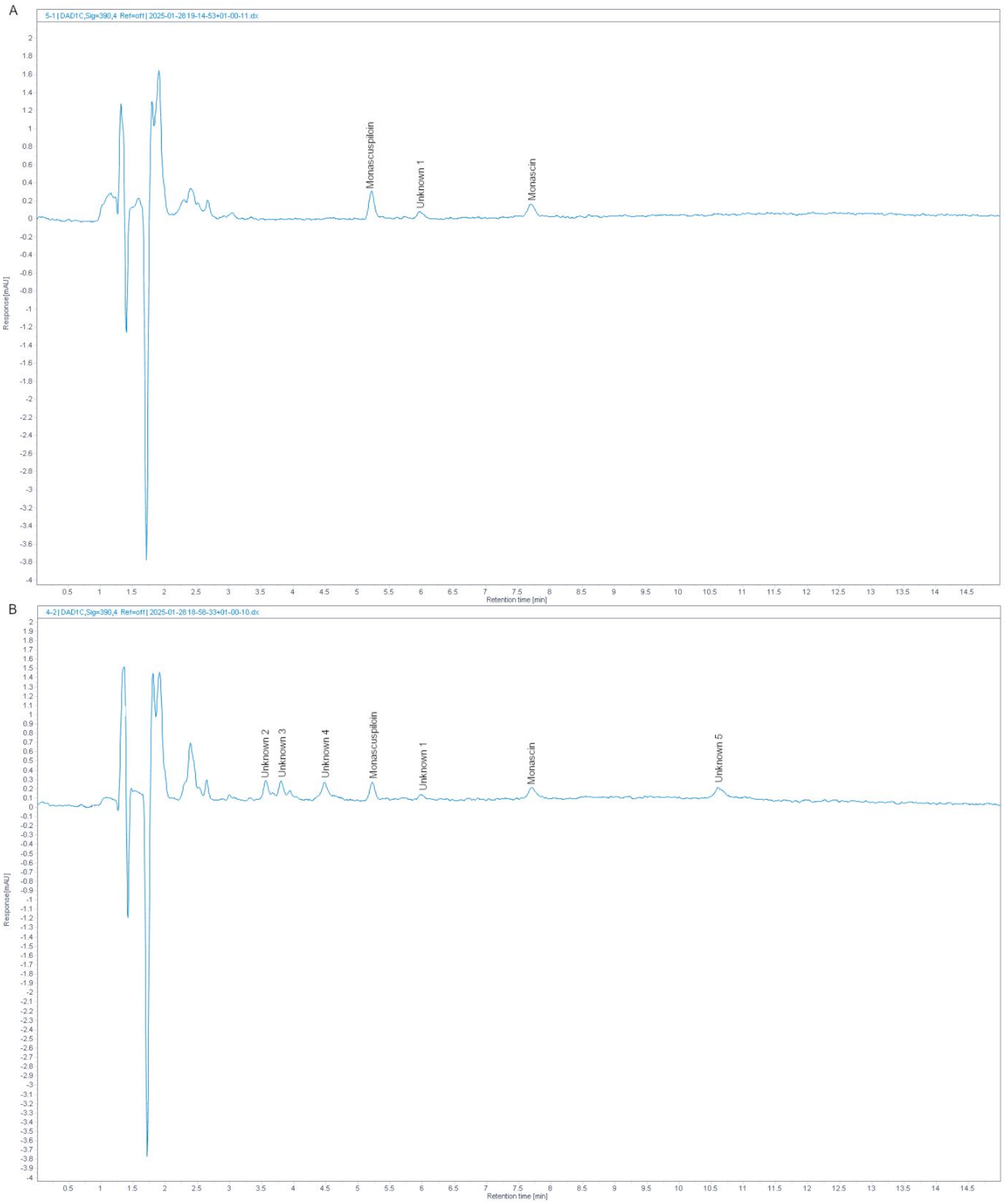

Figure S1. Typical chromatogram obtained by UHPLC analysis. DAD detector set up at 390 nm for yellow pigments detection. **A.** Extract from cultivation of *Monascus pilosus* with sucrose 100 g/L and tryptone 8.54 g/L. **B.** Extract from cultivation of *Monascus pilosus* with sucrose 50 g/L and tryptone 8.54 g/L with 50 g/L of NaCl. Detected pigments: Monascuspiloin with retention time 5.2 min; Monascin with retention time 7.7 min; and Unknown pigments.

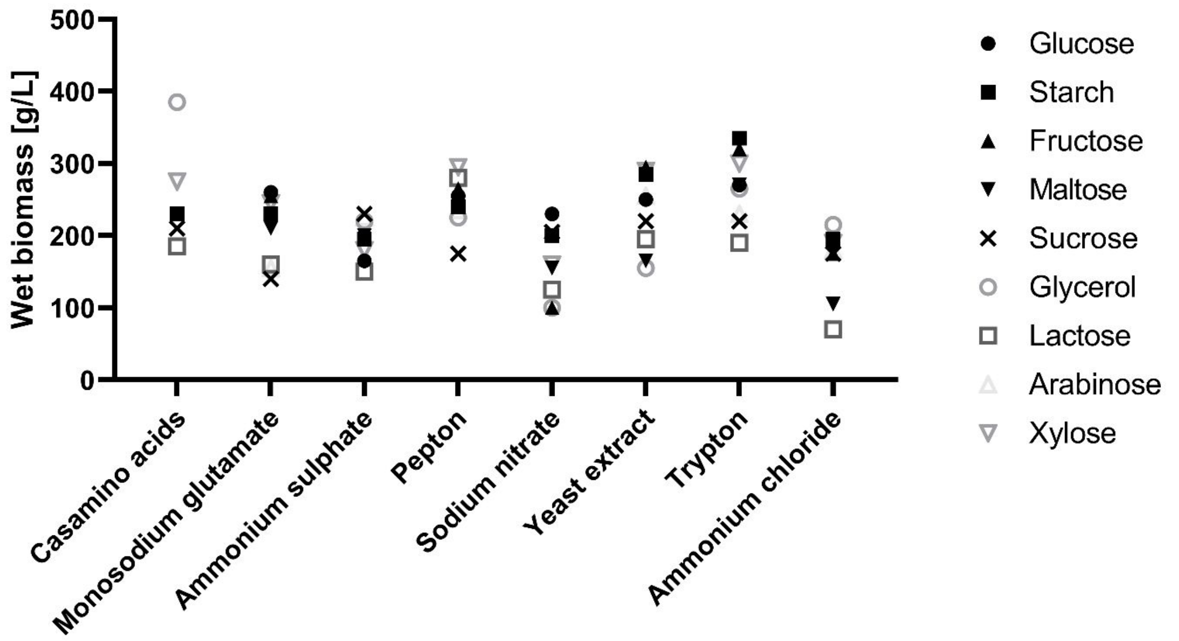

Figure S2. Wet biomass obtained from cultivations with different carbon and nitrogen sources.

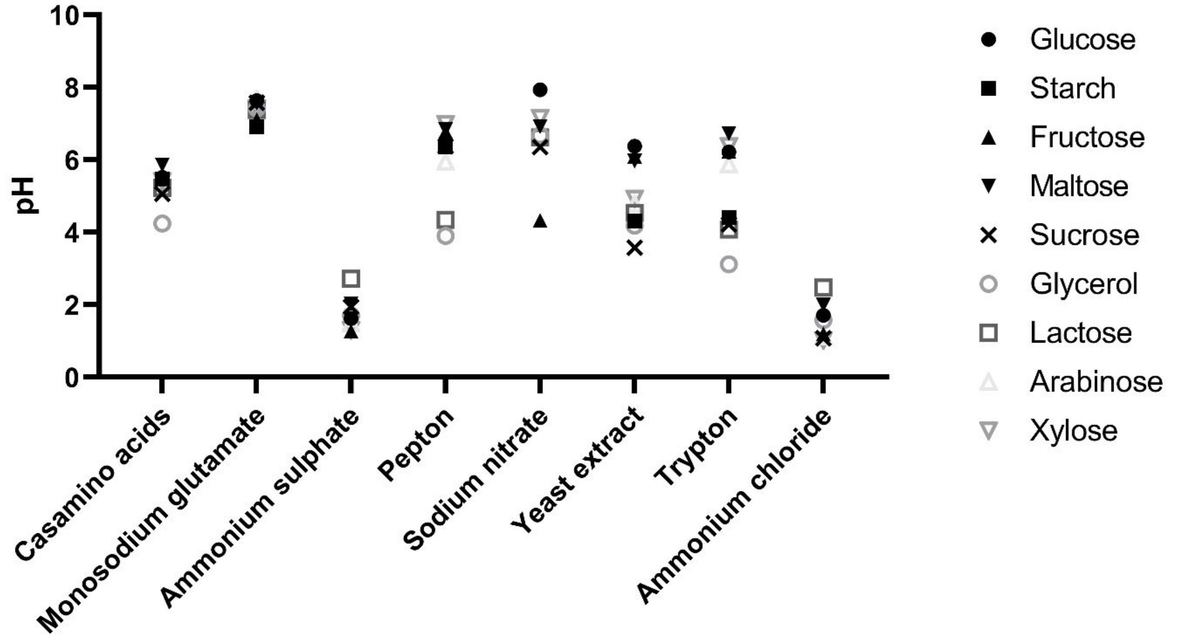

Figure S3. pH values of culture broth determined at cultivations with different sources of carbon and nitrogen sources.
